# An Unusual Intraoperative Transesophageal Echocardiographic Approach to Detect Acute Superior Vena Cava Syndrome in Mitral Valve Repair: A Case Report

**DOI:** 10.1155/cria/3966587

**Published:** 2025-10-15

**Authors:** Martina Bezzi, Matteo Nafi, Tiziano Cassina

**Affiliations:** ^1^ Division of Anesthesiology, Department of Acute Care Medicine, Geneva University Hospitals, Geneva, Switzerland, hug-ge.ch; ^2^ Department of Cardiac-Anesthesiology, Cardiocentro Ticino Institute, Ente Ospedaliero Cantonale, Lugano, Switzerland, eoc.ch

**Keywords:** cardiac surgery, color Doppler, pulsed-wave Doppler, superior vena cava stenosis, transesophageal echocardiography, transgastric view

## Abstract

The diagnosis of superior vena cava (SVC) syndrome is always challenging, even more so when it happens intraoperatively. An usual transgastric‐transesophageal projection allows for the visualization and quantification of caval flow acceleration, critical in leading to rapid diagnosis and quick reaction in the operative setting. We present the case of a patient who underwent successful mitral valve reconstruction and left atrial appendage closure via median sternotomy on cardiopulmonary bypass and, intraoperatively, developed iatrogenic SVC syndrome with hypotension, facial cyanosis, and conjunctival edema. We used an uncommon deep transgastric‐transesophageal echocardiographic projection, which proved crucial in promptly identifying SVC stenosis and in immediate intraoperative management. Our case report shows the efficacy of this dedicated deep‐transgastric view, which may be included as part of a routine echocardiographic exam in operations that require bicaval cannulation, leading to increased risk of acute SVC stenosis.

## 1. Introduction

Acute iatrogenic superior vena cava (SVC) syndrome is a rare but serious complication of cardiac surgery, characterized by inadvertent narrowing or obstruction of the SVC and cavoatrial junction, that may have detrimental and potentially life‐threatening clinical consequences.

Central venous catheter positioning or tight surgical suturing during cardiac procedures involving cardiopulmonary bypass (CPB) increases the risk of mechanical compression of the SVC, leading to insufficient drainage [1]. Early recognition and intervention are critical for ensuring patient safety [2–4].

Transesophageal echocardiography (TEE) plays a pivotal role in intraoperative diagnosis. TEE provides real‐time imaging and hemodynamic assessment of flow patterns and flow velocities, allowing immediate recognition and treatment of abnormalities that identify stenosis [5, 6].

We present the case of a patient who underwent elective mitral valve repair and left atrial appendage (LAA) closure and developed SVC stenosis caused by tight surgical suturing after SVC cannula removal. The condition was promptly diagnosed using an uncommon transgastric TEE projection, and immediate surgical intervention resolved hemodynamic instability.

## 2. Case Presentation

We obtained written informed consent from the patient for publication of this case report.

A 69‐year‐old female patient with severe mitral valve insufficiency of a myxomatous valve with prolapse of both leaflets was admitted for elective mitral valve repair and LAA closure via CPB via median sternotomy. The medical history revealed only arterial hypertension treated with an angiotensin receptor blocker.

Preoperative imaging, including transthoracic echocardiography (TTE), revealed severe mitral regurgitation due to bileaflet prolapse, preserved left ventricular systolic function, and severe dilation of the left atrium. TEE confirmed myxomatous degeneration of the mitral valve with bileaflet prolapse, resulting in severe eccentric mid‐to late‐systolic regurgitant jets; additionally it showed mild aortic and tricuspid regurgitation.

Upon arrival at the operating room, after the placement of standard anesthesia monitoring care tools including bispectral index (BIS) monitor and cannulation of the left radial artery for invasive blood pressure monitoring, uneventful intravenous induction was performed, followed by orotracheal intubation. An Arrow 3‐lumen 7 Fr central venous catheter was placed in the right internal jugular vein under ultrasound guidance without complications, and a TEE probe was placed to conduct a comprehensive TEE examination according to international guidelines [7].

After the administration of unfractionated heparin, venous cannulas were placed in the superior and inferior vena cava (bicaval cannulation), an arterial cannula was placed in the ascending aorta, and CPB was initiated. A 34 mm mitral ring was placed without leaflet resection, and the LAA was closed with surgical sutures. No surgical complications were reported, and CPB lasted for 80 min, with a cross‐clamping time of 68 min. TEE revealed correct LAA closure and mitral annuloplasty without paravalvular leakage and absence of valvular stenosis with an average pressure gradient of 5 mmHg; both right and left ventricular functions were preserved.

After administration of a heparin antagonist, the arterial and venous cannulas were removed, and vascular access was closed with surgical sutures. Shortly thereafter, the patient’s clinical condition gradually worsened, with hypotension, facial cyanosis, and mild edema of the conjunctivae, suggesting insufficient cervical venous drainage.

**Figure 1:**
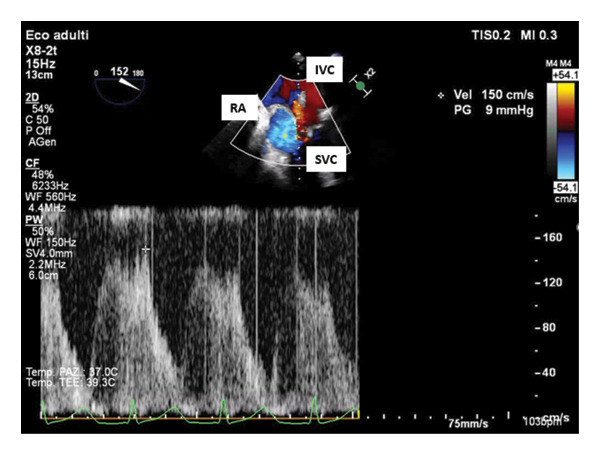
Deep transgastric pulsed wave color Doppler flow velocity in the acute superior vena cava stenosis. RA: right atrium; IVC: inferior vena cava; SVC: superior vena cava.

**Figure 2:**
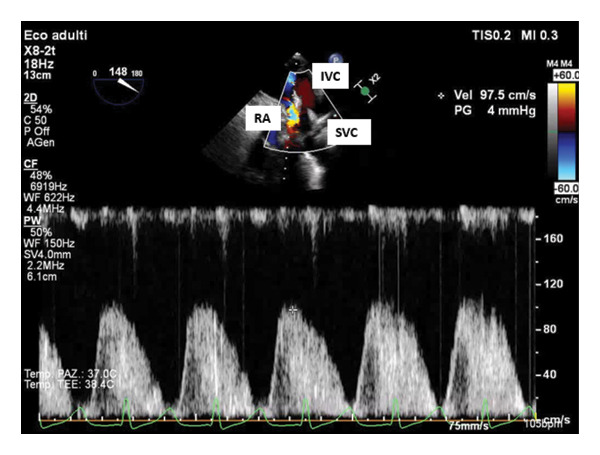
Deep transgastric pulsed wave color Doppler flow velocity after releasing the superior vena cava suture. RA: right atrium; IVC: inferior vena cava; SVC: superior vena cava.

To verify our hypothesis of SVC syndrome and to better quantify caval flow acceleration, we used a specific TEE deep transgastric view with the probe oriented toward the right at 120°–150°. In this projection, the SVC blood flow was parallel to the pulsed‐wave Doppler beam, allowing evaluation of the flow velocity, which was measured at 150 cm/s (Figure 1). After removal of the tight surgical suture and its replacement with a looser suture, the flow velocity significantly decreased to 97.5 cm/s (Figure 2). A 35% reduction in flow velocity, although still above the optimal range (10–45 cm/s) [8], reversed cyanosis and led to a significant hemodynamic improvement. At the end of the procedure, the patient was transferred to the intensive care unit and successfully extubated 2 h later. The following clinical course was uneventful.

## 3. Discussion

Acute SVC syndrome is a rare, but potentially fatal, complication of cardiac surgery. It is characterized by cervical venous congestion, reduced preloading, and cerebral edema. Common symptoms include dyspnea, orthopnea, cough, stridor, dysphagia, headache, nausea, syncope, blurred vision, and altered mental status. Common signs included facial and neck edema (82%), jugular turgor (63%), upper extremity edema, distention of the superficial thoracic veins, thoracic collateral circulation, cyanosis, altered neurological status, papilledema, stupor, hemodynamic instability, airway compromise, coma and death.

In our case, the removal of the venous cannula was marked by leakage of the SVC, which required a second suture. Several minutes later, the patient experienced increasing hypotension, which was initially attributed to hypovolemia, and was subsequently treated with crystalloid infusion. However, despite crystalloid and norepinephrine administration, the patient had persistent hypotension with evidence of facial cyanosis and conjunctival edema, although BIS monitoring did not show any modifications.

**Figure 3:**
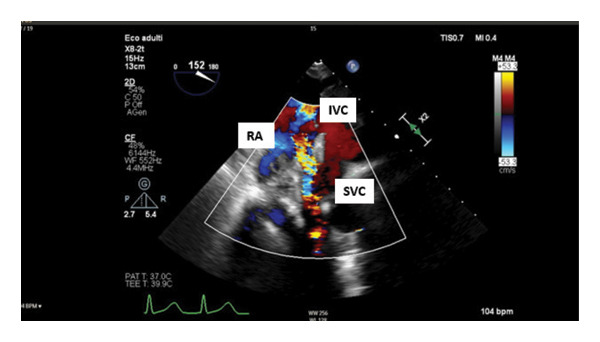
Deep transgastric color Doppler flow velocity in the setting of acute vena cava stenosis. RA: right atrium; IVC: inferior vena cava; SVC: superior vena cava.

To confirm this hypothesis, we used an uncommon transgastric TEE view (Figure 3). The standard mid‐esophageal bicaval projection allows only the assessment of qualitative acceleration through the color Doppler velocity, while this specific transgastric projection with the probe rotated to the right at 120°–150° allows the visualization and quantification of flow acceleration in the SVC with both continuous and pulsed wave Doppler. By providing detailed real‐time visualization, this unusual TEE projection significantly improves diagnostic accuracy and ultimately patient outcomes in complex cardiac surgery.

Although this projection is not part of a standard approach according to international guidelines, it takes advantage of classic transgastric projections without any increased iatrogenic risk.

However, some aspects of this case require careful discussion. To the best of our knowledge, there are no reference values for normal flow velocity in the SVC during mechanical ventilation via TEE, and the values reported by TTE refer to patients on spontaneous breathing ventilation [8–10]. Furthermore, because there is no validated TEE reference values for SVC flow velocities, clinical assessment remains crucial.

Compared with the preoperative values, greater intraoperative values of caval flow acceleration may aid in the diagnosis of SVC syndrome, especially in patients with less evident clinical signs.

## 4. Conclusion

In conclusion, the use of this specific deep transgastric‐TEE projection could be an adjunctive tool for early diagnosis and treatment of SVC syndrome during cardiac surgery requiring bicaval cannulation and should be routinely considered. However, there are currently no validated reference values for transesophageal measurements of blood flow acceleration in the SVC. Therefore, further studies are necessary to establish these parameters and fully integrate this imaging modality into clinical practice, especially for the most complex cases where SVC syndrome is suspected and quantification of SVC obstruction is required.

**Nomenclature**
SVC: Superior vena cava
CPB: Cardiopulmonary bypass
TEE: Transesophageal echocardiography
TTE: Transthoracic echocardiography
LAA: Left atrial appendage

## Ethics Statement

As a single‐case report with the patient’s signed consent, no other ethical review was required.

## Consent

We obtained written informed consent from the patient for publication of this case report.

## Disclosure

Please note that an earlier version of this article was published as a pre‐print with this reference: Martina Bezzi, Matteo Nafi, Tiziano Cassina. A Novel Intraoperative Transesophageal Echocardiographic Approach to Detect Acute Superior Vena Cava Syndrome. Authorea. October 29, 2024. DOI: 10.22541/au.173017636.68508494/v1.

I confirm that our article has never been accepted or published by another scientific journal but only as a pre‐print. Furthermore, compared to the version published as a pre‐print, we have implemented changes that, in my opinion, make the suggestions in this case report even more expendable in clinical practice.

## Conflicts of Interest

The authors declare no conflicts of interest.

## Author Contributions

Martina Bezzi (corresponding author): substantial contributions to the conception, writing, editing and revision of the work.

Matteo Nafi: substantial contributions to the conception, writing and revision of the work.

Tiziano Cassina: revision of the work.

## Funding

The authors received no specific funding for this work.

## Data Availability

The data used in this article are available upon request from the corresponding author.
